# Mind and Matter

**Published:** 1858-11

**Authors:** 


ART. V.—*Mind and Matter: or Physiological Inquiries. In a Series of Essays intended to Illustrate the Mutual Relations of the Physical Organization and the Mental Faculties.* By Sir Benjamin Brodie, Bart., D. C. L., Vice-President of the Royal Society. With Additional Notes, by an American Editor. New York: Samuel S. & William Wood, 389 Broadway. 1858.

We have few ornaments to the literature of our profession like the book before us. The dry detail of statistically established facts, are seldom relieved but by the verbose and entangled speculations of the merely theoretical enthusiast. Elegance of style, also, is little cultivated by medical writers, so that even authors of eminence are contented with a hasty and imperfect sketch of their vast experience, and more pleasing writers are apt to seduce us into false and untenable theories. It is most frequently our fortune to have the good presented to us with the disadvantages of an arid, curt, and frequently careless style, not unfrequently burdened with an enormous mass of cases which the statistical fever of the day has led some to consider absolutely necessary. Not that we would be understood as underrating the value of statistical inquiry, and as not appreciating the vast strides which medical science has made by means of the efforts of the great minds which have been turned to this mode of investigation: all this we admit, but it must also be admitted that the mind will tire of this "concentrated nourishment," and occasionally desire something which is pleasing as well as instructive. As the body needs repose, so does the mind; and the seductive avenues of thought opened to us by the distinguished author of this little volume refresh the intellect, like a sojourn among the hills and trees, removed from the active cares of life, and surrounded only by nature. No subject could have been better chosen than "*mind and matter*;" that which has the most of the incomprehensible and the beautiful; and by no one could it be more ably and pleasingly handled than by Sir Benjamin Brodie.

The graceful style of the author is admirably calculated to carry out the method of dialogue which has been adopted in this work. It is supposed to be series of familiar conversations between three friends, two of whom are visiting the other who has retired from active life to spend the rest of his days in the country.

"Ergates" at the end of the "London season," meets his friend Crites, who, just released from the duties of the bench, proposes to pay a visit to Ebulus, the mutual friend of their boyhood, who has retired from the active duties of an official position and gone to reside in the country, a hundred miles from the metropolis. Here the three friends indulge in scientific reflections suggested by their position and the things around them. The mind, with a discussion of some of its attributes, and the influence of education upon its character, form the subject of the first dialogue.

The occupations of men are so diverse, and habit renders every one so dependent for happiness upon the prosecution of his own pursuits, whatever they may be, and upon the attainment of his own ends, that unreflecting minds do not appreciate the labor of others. The mechanic often thinks the fee of the professional man too easily earned; all men regard a pittance as sufficient compensation for the literary man, as is sufficiently proven by his notorious poverty; and few appreciate that a few hours of close mental application are more exhausting than treble the amount of bodily exertion, unaccompanied by any great mental exercise.

These are facts which we can readily appreciate, and which are exemplified in the history of all men who have been subjected to severe mental exertion. We quote the author's instance of Lord Bacon.

"But at every step in the composition of his philosophical works, Lord Bacon had to think; and no one can be engaged in that which requires a sustained effort of thought, for more than a limited period of the twenty-four hours. Such an amount of that kind of occupation must have been quite sufficient, even for so powerful a mind as that of Lord Bacon. Mental relaxation after severe mental exertion, is not less agreeable than bodily repose after bodily labor. A few hours of *bonâ fide* mental labor daily, will exhaust the craving for active employment, and will leave the mind in a state in which the subsequent leisure (which is not necessarily mere idleness) will be as agreeable as it would have been irksome and painful otherwise."

This is undoubtedly true; the leisure after a severe mental tension has nothing of the *ennui* which is the invariable lot of an indolent man. After the mind has been actively and powerfully exerted even for a short time, the repose which follows is as delicious as rest after severe corporeal exercise. The hard working man does not feel the necessity for the exciting amusement which is almost indispensable to the men whose only aim is to kill time. The soothing influence of the quiet of the country is frequently particularly refreshing to a mind habituated to severe exertion; while the unfortunate "man about town" cannot endure it unless enlivened by hunting, fishing, or some exciting occupation.

The thoughts of the author in regard to this subject are exceedingly correct and beautiful, and the examples by which they are illustrated, such as to impress the reader with their force.

"Sir Walter Scott," he says, "describes himself as having devoted about six hours daily to literary composition, and his mind was then in a state to enjoy some lighter pursuits afterwards. After his misfortunes, however, he allowed himself no relaxation, and there can be little doubt that this over-exertion contributed, as much as the moral suffering which he endured, to the production of the disease of the brain which ultimately caused his death. Sir David Wilkie found that he was exhausted if employed in his peculiar line of art for more than four or five hours daily."

But we must repress our inclination to follow this line of argument, and pass on to another branch of the subject. This is a consideration of deceptions and delusions. The author truly says, in speaking of the proneness of the world to be led into delusions,

"That a great extension of education and knowledge does not produce any corresponding improvement in this respect. Still, in the end, good sense prevails. Errors and deceptions last only for a time. Those which disgrace one age banish, and are succeeded by those which disgrace the next. But a truth once established remains undisputed, and society, on the whole, advances."

There is no subject which could be more interesting to us as medical men than this. We have been selected by Providence to feel the weight of nearly all the impositions which afflict the world. The horde of homœopathists, hydropathists, Thompsonians, natural bone setters, clairvoyants, spirit-rappers, *et id omne genus* which infest society, indicate to *us* most forcibly, the unbounded credulity of the masses. Still, as our author remarks, one deception only gives way to another; humbug is a many headed hydra, and the rapidity of their reproduction rather discourages us in our efforts to lop them off. We generally find that people who are devoted to one system of delusion, when they are deprived of that, do not come back to common sense, but lapse into another. Most of our spiritualists are homœopathists, or patrons of some other quack system. It seems, indeed, as if there were certain persons who *must* be deluded, and we are not sure but that the wisest course for us to pursue would be to let them alone.

The first dialogue is here ended, and we are led in the second, by easy transition, to a discussion of the subject of natural theology, some of the offices of the nervous centres, and memory. Memory is one of the most curious and iuexplicable phenomena of the mind. What change is it which takes place in the nervous centres, by which we are enabled to recall past events? That the intellect is seated in the cerebral hemispheres, there can be no doubt, and memory is certainly one of the most important attributes of the intellect. The phenomena which are presented in certain morbid conditions are exceedingly interesting. Memory is inseparably allied with all the other mental phenomena; and their natural operations being so little understood, it follows that deviation from healthy action are proportionally obscure; thus the subject of insanity, though deeply interesting, can be carried but to a certain point and no farther. The author relates some interesting instances of the effect upon the memory of blows on the head.

"A groom in the service of the Prince Regent was cleaning one of some horses sent as a present to his Royal Highness by the Shah of Persia. It was a vicious animal, and she kicked the groom on the head. The groom did not fall, nor was he at all stunned or insensible; but he entirely forgot what he had been doing at the moment when the blow was inflicted. There was an interval of time, as it were, blotted out of his recollection. Not being able to account for it, he supposed that he had been asleep, and said so to his fellow servants, observing at the same time " that he must set to work and clean the horse, which he had neglected to clean in consequence of his having fallen asleep."

Instances of this kind might be multiplied; they come under the observaion of every physician, though not perhaps as strongly marked as the case just related. Prof. Hamilton, we recollect, was at one time making inquiries into this very matter, of persons who had been rendered insensible by injuries to the head. We believe that the result of his investigations never have been published, and can merely state from recollection some of the answers which he received. Most persons stated that they retained no impression of what occurred some seconds before the injury. For example, persons who have fallen from a height did not remember in what manner the accident occurred; and it is doubtless the fact that they would have remembered the circumstances if they had received no serious injury. Fevers, attacks of apoplexy, and some other disorders, occasionally produce curious effects upon the memory. A patient is referred to who lost the power of reading, after a attack of fever.

"Although in looking at a book he recognized the letters of the alphabet, he forgot what they spelled, and was under the necessity of learning again to read. Nevertheless, he knew his family and friends; and his judgment, when the facts were clear in his mind, was perfect."

This interesting subject is continued in the third dialogue: "The sequence and association of ideas;" "suggestion of ideas by internal causes acting on the brain by the nerves, or through the medium of the blood;" the influence of narcotics, morbid poisons, lithic acid, impure atmosphere, and other physical agents on the condition of the mind, are here discussed; the reflections of the opium eater; the sensations of the dyspeptic and gouty sensualist; the drunkard; the "Malay under the influence of the East Indian hemp," are depicted; but we are compelled to pass over this as well as many other interesting passages. These reflections lead again to "*illusions*." By the author's theory, supported by examples, we are able to account for some of the remarkable things which persons, credible in other matters, assert that they have actually seen; especially the idea that they are able to see and converse with departed friends. It is impossible, in some instances, to suppose that the narrators of these wonderful tales are attempting to deceive us, and our only theory is that they are themselves deceived. Instances are related by the author, which are not less marvelous than those soberly related to us by our most credulous and enthusiastic votaries of spiritualism. The following is one of the most remarkable instances of illusion, and what is as remarkable, the individual himself was convinced that it was an illusion.

"A gentleman, eighty years of age, had been for some time laboring under hypochondriasis, attended with some other indications of cerebral disease. On a cold day in winter, while at church, he had a fit which was considered to be apoplectic. He was taken home and bled, and recovered his consciousness, not being paralytic afterwards. He died, however, in a few days after the attack. During this interval, though having the perfect fect use of his mental faculties, he was haunted by the appearance of men and women, sometimes in one dress, sometimes in another, coming into and loitering in the room. They were so distinct that, at first, he always mistook them for relatives, and wondered that his family should have allowed such persons to intrude themselves upon him. But he soon, by a process of reasoning, corrected this error, and then talked of them as he would of the illusions of another person. You have probably read the history of Nicolai, the bookseller of Berlin, who was haunted by visions of persons coming into his apartment, sitting down, and even conversing with him and with each other, and this during a period of several months. He also was at first taken by surprise, believing the phantoms to be real objects; but was soon enabled to convince himself that they were not so. His recovery was attributed to an improved state of his bodily health."

With such instances as these, and numerous others upon record, is it remarkable then that persons should imagine that they have seen and conversed with absent friends, and receive as an explanation that their spirits had revisited the earth? These discussions, however, are profitless; it requires no argument to convince a physiologist that spiritual manifestations are illusions, though their study, in a psychological point of view, is interesting.

The rapidity with which events are presented to the mind, under certain circumstances, is exceedingly remarkable. This point does not escape comment in the volume before us. Under certain circumstances, particularly in drowning, the mind appears to take cognizance almost immediately of every act of a past life. The experience of Sir John Barrow, in this regard, is here quoted from his autobiography. When he was preserved from being drowned, he says:

> "Every incident of his former life seemed to glance across his recollection in retrograde succession, not in mere outline, but the picture being filled with every minute and collateral feature, forming a kind of panoramic view of his entire existence, each act of it accompanied by a sense of right and wrong."

This is by no means an extraordinary experience, and it happened indeed to ourselves under the same circumstances. We were submerged for not more than a few seconds, and every act which we had been taught to consider wrong appeared in detail. This happened at the age of about twelve years, but we have now the most vivid recollection of our sensations at that time. Almost every one is struck with the rapidity with which a dream, the incidents of which would occupy an hour or so, to write down in detail, is conceived by the mind. We fall asleep, have a dream of great length, and on waking find that we have been asleep scarcely a moment. It happened to the late Lord Holland to have an opportunity of measuring the length of time in such a case.

> "On an occasion, when he was much fatigued, while listening to a friend who was reading aloud, he fell asleep, and had a dream, the particulars of which would occupy him a quarter of an hour or longer to express in writing. After he awoke, he found that he remembered the beginning of one sentence, while he actually heard the latter part of the sentence immediately following it, so that probably the whole time during which he had slept did not occupy more than a few seconds."

Our author then considers intelligence and instinct, and thinks very correctly, as we conceive, that intelligence is not peculiar to man, nor instinct to the lower animals. The conversations on this subject are exceedingly interesting and instructive, but we have not the space to give our readers a review of them, and must refer them to the volume itself.

We have now attained, and indeed, exceeded the limits within which we felt compelled to confine ourselves, but we cannot close our review without mentioning the subjects of the sixth and last dialogue. This is devoted to the science of human nature. In the study of human nature all are interested; here we have no distinction of profession or pursuits, we all wish to know our fellow-men. The greatest men of all ages have risen to their eminence by their knowledge of men, and our author says that the great instrument of advancement possessed by such men as Cromwell or Napoleon is,

> "That he cannot have risen by his own exertions through the various grades which he has occupied in the course of his career, associating with others on equal terms, without acquiring an insight into men's minds and characters, which it would not have been possible for him to have acquired elsewhere. The unhappy Louis XVI, and Marie Antoinette, surrounded as they had been by the etiquette, and misled by the adulations of a Parisian Court, received their first lessons in human nature from the brutal frenzy of a revolutionary mob."

The dialogue closes with a familiar discussion of phrenology which we have not the space to follow.

It is rare, as we before remarked, to find a book like this, written by an eminent member of the profession; and we can justly be proud of this little work, which is as interesting to the general as to the professional reader, and which presents in the most seductive possible form, well established truths, which should be known by every enlightened man. Facts, and especially physiological facts, are not often presented to the general reader in a fascinating form; but here one would imagine that he was receiving instruction in a familiar talk with a brilliant conversationalist. Such is the book of which we have endeavored to give an idea in the preceding review, and as such we recommend it to be read by all, physicians and laymen.*

---

* This work is sent by mail by S. S. & W. Wood, 389 Broadway, New York, free of postage. Price $1.

